# Utilization of Hypolipidemic Drugs, Patterns, and Factors Affecting Dyslipidemia Among Type 2 Diabetes Mellitus at a Tertiary Care Teaching Hospital in South India

**DOI:** 10.7759/cureus.34748

**Published:** 2023-02-07

**Authors:** Sandeep Khot, Ananya Chakraborty, Savitha Vijaykumar

**Affiliations:** 1 Pharmacology and Therapeutics, Vydehi Institute of Medical Sciences and Research Centre, Bengaluru, IND

**Keywords:** lipid profiles, diabetes type 2, dyslipidemia, statins, drug utilization

## Abstract

Background

The prevalence of dyslipidemia is higher in type 2 diabetes mellitus (T2DM) and hypolipidemic drugs like statins are effective for the primary and secondary prevention of cardiovascular events. Most of the patients with type 2 diabetes have a mixed type of dyslipidemia. This study aimed to evaluate the utilization of hypolipidemic drugs, patterns, and factors affecting dyslipidemia in T2DM participants.

Methods

This cross-sectional observational study was approved by the institutional ethics committee (IEC) of the Vydehi Institute of Medical Sciences and Research Center. It was conducted for a period of one year from July 2021 to June 2022. Participants with T2DM visiting the Department of General Medicine and Endocrinology were enrolled after obtaining informed consent. Demographic details, medication history, and laboratory data were recorded in case report form and statistical measures were applied.

Results

Out of 237 participants enrolled in the study, the predominance (n=133, 56%) was males. The mean age of the study population was 47.92±9.17 years, and the mean duration of diabetes was 6.8±5.3 years. Out of the total participants, 164 (69%) had deranged lipid profiles. Out of them, 129 (78.65%) were on hypolipidemic drugs. Regarding drug utilization, 122 (94.6%) received statins either rosuvastatin (54%) or atorvastatin (40%). In the deranged lipid profiles pattern, 24% (58) participants had one abnormal lipid parameter and the majority 70% (166) had combined lipid profile abnormality. Factors like increased BMI were significantly associated with dyslipidemia (p=0.004). Utilization of hypolipidemic drugs was also significantly associated with the control of dyslipidemia (p<0.001). It was observed that participants who were not on lipid-lowering drugs had 5.38 times more chance of dyslipidemia (OR=5.38; CI=2.82-10.28; p<0.001).

Conclusion

A high prevalence of dyslipidemia was observed among diabetic patients. Statins were the most prescribed drug in the study. BMI and lack of pharmacotherapy were found to have a statistically significant association with dyslipidemia in diabetic patients.

## Introduction

Diabetic dyslipidemia is characterized by elevated plasma triglyceride levels, low high-density lipoprotein cholesterol (HDL-C) levels, and an increase in several small dense forms of low-density lipoprotein (LDL) particles which are highly atherogenic [1]. According to ICMR guidelines for the management of type 2 diabetes mellitus (T2DM), targets for lipid control in individuals are total cholesterol less than 200 mg/dL, HDL cholesterol more than 40 mg/dL for men, and more than 50 mg/dL for women, LDL cholesterol less than 100 mg/dL, triglycerides less than 150 mg/dL, respectively [2]. Dyslipidemia and hypertension are definite risk factors for atherosclerotic cardiovascular disease (ASCVD). ASCVD is also defined as coronary heart disease (CHD), cerebrovascular disease, or peripheral arterial disease and is the leading cause of morbidity and mortality in diabetics [3].

Besides antidiabetic drugs, lifestyle modification and hypolipidemic drugs are effective [3,4]. Statin is one such medication that has demonstrated efficacy in extensive studies and is advised in current guidelines for individuals with T2DM. In both primary and secondary prevention, statin showed a consistent reduction in mortality and major adverse cardiovascular events by (Risk ratio=0.80; 95% CI=0.77-0.83) for every one mmol/L (38.7 mg/dL) decrease in LDL-C [5]. American Diabetes Association (ADA) published medical care standards, including recommendations for starting lipid-lowering therapy and prescribing moderate-intensity statins for those with no additional risk factors. High-intensity statins are recommended for those with either CVD risk factors or overt CVD with consideration of 10-year ASCVD risk [3]. The American College of Cardiology/American Heart Association (ACC/AHA) clinical practice guidelines advise prescribing moderate-intensity statins to patients aged 40 to 75 who have T2DM and those with LDL-C more than 70 mg/dL without considering 10-year ASCVD risk [6]. According to ADA high-intensity statin therapy like atorvastatin (40-80mg) and rosuvastatin (20-40mg) lowers LDL cholesterol by 50% and moderate-intensity statin therapy like atorvastatin (10-20mg), rosuvastatin (5-10mg), simvastatin (20-40mg), pravastatin (40-80mg), lovastatin (40mg), fluvastatin extended-release (80mg), pitavastatin (1-4mg) lowers LDL cholesterol by 30%-49% [3].

Previous studies on diabetic dyslipidemia in India found that dyslipidemia was more in females and the age group 45-54 years. They also concluded that the majority 44.2% of participants suffer from a mixed type of dyslipidemia [7,8]. They also found that in India the prevalence of dyslipidemia is about 86% [8]. Only a few studies were conducted in the southern part of India. This current study focuses on statin utilization in people with diabetic dyslipidemia.

## Materials and methods

The present study was a cross-sectional observational study. The study was conducted by the Department of Pharmacology in collaboration with the Department of Endocrinology and the Department of General Medicine at Vydehi Institute of Medical Sciences and Research Center, Bengaluru. The duration of the study was one year from July 2021 to June 2022. The study was conducted after receiving approval from the institutional ethics committee, under the registration number VIEC/PG/APP/015/2020-21. Participants were screened for inclusion and exclusion criteria. Both male and female patients with type 2 diabetes irrespective of lipid-lowering therapy between 18 and 60 years attending the OPD and consenting to the study were included. The exclusion criteria included type 1 diabetes mellitus patients, gestational diabetes mellitus, and inpatients. The study objectives and process were explained to the participants or their relatives in their language. Participants were asked to read and sign an informed consent form. The demographic details, medical history, personal history, medication history, and laboratory data of all participants included in the study were recorded on Case Report Form (CRF). Participants were divided into two groups based on lipid profile level. Group one was participants with high lipid levels than the normal range, and group two was participants with low lipid levels than the normal range. Drug utilization was evaluated. Several factors like age, gender, body mass index (BMI), duration of disease, comorbidities, drug utilization of hypolipidemic drugs, family history, and HbA1c were compared between the two groups. Finally, all details from the CRF form were recorded on an excel sheet and statistical measures were applied. The following biological references were considered low lipid levels. Total cholesterol less than 200 mg/dL, triglycerides less than 150 mg/dL, HDL cholesterol more than 40 mg/dL, LDL cholesterol less than 100 mg/dL, VLDL between 2 and 30 mg/dL. Any one or more than one lipid parameter higher than the normal lipid range was considered high lipid levels. The sample size was calculated considering a confidence interval of 95%, precision of 5%, and power of study at 80%.

Data collected were entered into a Microsoft office excel sheet. Demographic details were subjected to descriptive statistical analysis and were expressed as mean SD and percentages. Drug utilization patterns were analyzed and expressed in frequencies and percentages. The categorical variable was compared using the chi-square test and odds ratio. Association between all categorical variables and lipid profile was observed and a P-value of less than or equal to 0.05 was considered statistically significant.

## Results

Demographic details

The total number of participants in the study was 237. Out of 237 participants, 56% (133) were males. Out of the males, 72% (96) were found to have high lipid levels. The mean age (± SD) of the participants was 47.91 ± 9.17 years. Most participants were in the age group of more than 30 years. The mean BMI (± SD) was 25.27 ± 4.08 kg/m^2^. Most participants had a family history of T2DM. The mean duration (± SD) of the disease was 6.8 ± 5.3 years. Most participants had a duration of disease of less than six years. And in the same category, 75% (91) were found to have dyslipidemia. The mean HbA1c (± SD) was 8.9 ± 2.35%. The results are presented in Table 1.

**Table 1:** Demographic details of study participants BMI= Body Mass Index, HbA1c= Glycated hemoglobin

Variables	Category	Mean ± SD	n (%)	Dyslipidemia	
				High lipid levels n (%)	Low lipid levels n (%)
Sex	Male	-	133 (56)	96 (72)	37 (28)
	Female		104 (44)	68 (65)	36 (35)
Age	< 30 years	47.91 ± 9.17	7 (3)	7 (100)	0 (0)
	> 30 years		230 (97)	157 (68)	73 (32)
Occupation	Merchants	-	51 (22)	40 (78)	11 (22)
	Farmer		20 (8)	15 (75)	5 (25)
	Employee		67 (28)	42 (63)	25 (37)
	Homemakers		99 (42)	67 (68)	32 (32)
BMI	< 24.9 kg/m2	25.27 ± 4.08	106 (45)	62 (58)	44 (42)
	25-29.9 kg/m2		102 (43)	81 (79)	21 (21)
	≥ 30 kg/m2		29 (12)	21 (72)	8 (28)
Family history of diabetes mellitus	Yes	-	137 (58)	92 (67)	45 (33)
	No		100 (42)	72 (72)	28 (28)
Duration of diabetes mellitus	< 6 years	6.84 ± 5.30	122 (51)	91 (75)	31 (25)
	6-10 years		67 (28)	44 (66)	23 (34)
	> 10 years		48 (21)	29 (60)	19 (40)
HbA1c	≤ 7%	8.9 ± 2.3	61 (26)	43 (70)	18 (30)
	>7%		176 (74)	121 (69)	55 (31)
Hypertension	Yes	-	100 (42)	71 (71)	29 (29)
	No		137 (58)	93 (68)	44 (32)
Hypolipidemic drug utilization	Yes	-	129 (54)	108 (84)	21 (16)
	No		108 (46)	56 (52)	52 (48)

Hypolipidemic drugs utilization

**Figure 1:**
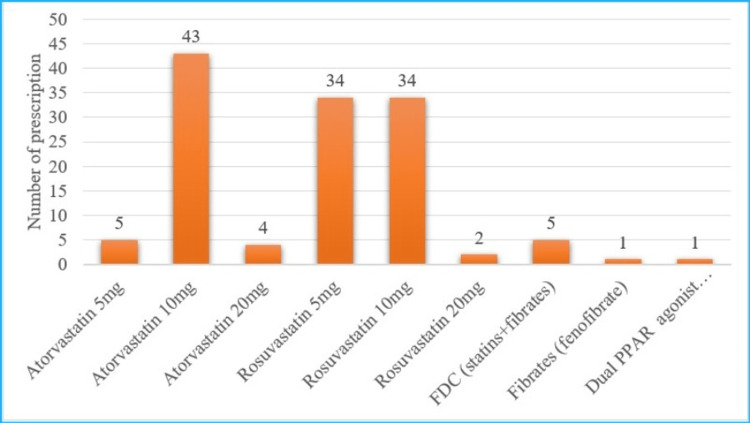
Frequency of hypolipidemic drugs prescribed FDC= Fixed Dose Combination, PPARs= Peroxisome proliferator-activated receptors

Out of 237 participants, 69% (164) participants were found to have high lipid levels. Out of them, 78.65% (129) received hypolipidemic drugs. A total of 129 generic drugs were prescribed. Most of the participants 94.6% (122) received HMG-CoA reductase inhibitors, statins. In fixed dose combination (FDC), rosuvastatin and fenofibrate was the common prescription. The drug utilization pattern is presented in Figure 1.

Patterns of dyslipidemia

Regarding dyslipidemia, high lipid level was observed in 69% (164) participants, isolated in 24% (58) of participants and combined abnormality in 70% (166). After analyzing dyslipidemia components low level of HDL-C was found in 35% (82), hypertriglyceridemia in 44% (104), elevated level of LDL in 60% (142), and hypercholesterolemia in 26% (61) participants, respectively. The details are presented in Table 3.

**Table 2:** Patterns of lipid abnormality among participants with T2DM HDL= High density lipoprotein TG= Triglycerides LDL= Low density lipoprotein TC= Total cholesterol

Lipid abnormality		n (%)
Isolated	Low HDL	18 (7.59)
	High TC	0 (0)
	High LDL	31 (13)
	High TG	9 (3.79)
Combined	Low HDL & High LDL	15 (6.3)
	Low HDL & High TG	14 (5.9)
	Low HDL, High LDL & High TG	21 (8.8)
	Low HDL, High LDL & High TC	1 (0.42)
	Low HDL & High TC	14 (5.9)
	High LDL & High TG	15 (6.3)
	High TG & High TC	1 (0.42)
	High LDL, High TC & High TG	32 (13.5)
	Low HDL, High LDL, High TC & High TG	13 (5.4)
Combined & Isolated	Low HDL	82 (35)
	High TG	104 (44)
	High LDL	142 (60)
	High TC	61 (26)

Factors affecting dyslipidemia

The factors like gender, age, occupation, hypertension, family history of diabetes, and duration of disease, HbA1c had no statistically significant association with dyslipidemia. The participants with hypertension had 1.2 times more chance of having dyslipidemia (OR=1.2; CI=0.60-2.38; p=0.59). The participants with a family history of T2DM had 1.17 times more chance of having dyslipidemia (OR=1.17; CI=0.62-2.20; p=0.62). Regarding BMI and dyslipidemia statistical significance was seen (p=0.004). It was seen that participants in the overweight and obese category had 1.07 times more chance of having dyslipidemia than those in the normal weight category (OR=1.07; CI=0.98-1.16; p=0.92). Regarding drug utilization and dyslipidemia statistical significance was observed (p<0.001). It was observed that participants who were not on lipid-lowering medication had 5.38 times more chance of having a derranged lipid profile (OR=5.38; CI=2.82-10.28; p<0.001). The results are presented in Tables 3, 4.

**Table 3:** Factors associated with dyslipidemia * denotes statistically significant difference.

Variables	Category	High lipid levels n (%)	Low lipid levels n (%)	P value	X^2^	
						Sex	Male	96 (72)	37 (28)	0.26	1.26	
Female	68 (65)	36 (35)									
Age (years)	< 30	7 (100)	0 (0)	0.07	3.21	
	> 30	157 (68)	73 (32)			
Occupation	Merchants	40 (78)	11 (22)	0.25	9.03	
	Farmer	15 (75)	5 (25)			
	Employee	42 (63)	25 (37)			
	Homemakers	67 (68)	32 (32)			
BMI (kg/m2)	< 24.9	62 (58)	44 (42)	0.004*	10.8	
	25-29.9	81 (79)	21 (21)			
	≥ 30	21 (72)	8 (28)			
Hypertension	Yes	71 (71)	29 (29)	0.60	0.26	
	No	93 (68)	44 (32)			
Family history of diabetes mellitus	Yes	92 (67)	45 (33)	0.425	0.63	
	No	72 (72)	28 (28)			
Duration of diabetes mellitus (years)	< 6	91 (75)	31 (25)	0.15	3.79	
	6-10	44 (66)	23 (34)			
	> 10	29 (60)	19 (40)			
HbA1c	≤ 7%	43 (70)	18 (30)	0.79	0.06	
	>7%	121 (69)	55 (31)			
Hypolipidemic drug utilization	Yes	108 (84)	21 (16)	<0.001*	28.1	
	No	56 (52)	52 (48)			

**Table 4:** Factors and correlation with dyslipidemia OR= Odds ratio, CI= Confidence interval * denotes statistically significant difference.

Variables	Category	High lipid levels n (%)	Low lipid levels n (%)	OR (95% CI)	P value	
						BMI (kg/m2)	< 24.9	62 (58)	44 (42)	1.07 (0.98-1.16)	0.92	
25-29.9	81 (79)	21 (21)									
≥ 30	21 (72)	8 (28)									
Hypertension	Yes	71 (71)	29 (29)	1.2 (0.60-2.38)	0.59	
	No	93 (68)	44 (32)			
Family history of diabetes mellitus	Yes	92 (67)	45 (33)	1.17 (0.62-2.20)	0.62	
	No	72 (72)	28 (28)			
HbA1c	≤ 7%	43 (70)	18 (30)	1.00 (0.89-1.14)	0.98	
	>7%	69 (121)	55 (31)			
Hypolipidemic drug utilization	Yes	108 (84)	21 (16)	5.3 (2.82-10.28)	<0.001*	
	No	56 (52)	52 (48)			

## Discussion

The purpose of this study was to evaluate the prevalence of dyslipidemia and the utilization of hypolipidemic drugs use among participants suffering from T2DM. In this study, 56% (133) of the participants were males, and high lipid levels were present in 72% (96) of males. Comparable results were observed in a prior study by Bekele et al. where 53.6% (120) participants were males and 70% (84) had high lipid levels [9]. In this study, 97% (230) of participants were older than 30 years of age, and 68% (157) of them had high lipid levels. Comparable results were observed by Bekele et al. where the majority of participants 66.5% (149) were older than 30 years and out of that 43.8% (98) had dyslipidemia. Kassahun Haile et al. where most participants 86.3% (214) were older than 30 years out of which 72.4% (155) had dyslipidemia [9,10]. The mean BMI in this study was 25.27 ± 4.08 kg/m2. The majority of participants 45% (106) had a BMI of less than 24.9 kg/m^2^ and 58% (62) of them had high lipid levels. Equivalent results were observed by Bekele et al. where 58% (130) participants were in a normal BMI category and 29.5% (66) had dyslipidemia [9]. These results contrasted with the study by Kebede et al. where 56% (183) were in the overweight category out of which 59% (113) had dyslipidemia [11]. This may be because due to different ethnicity. The majority of participants 51% (122) had a duration of disease of fewer than six years and among them 75% (91) had dyslipidemia. Similar results were observed by Hyassat et al. [12].

In the present study, 54% (129) of participants received hypolipidemic medications. Out of which 94.6% (122) of participants received statins, and atorvastatin accounted for 40% (52) prescriptions. Like Jayaram's study [13], Gupta et al. also found statins were prescribed in the majority of participants, and atorvastatin was mostly prescribed medication [14]. Similar results were observed in Patel's [15] and Adhikari's studies where atorvastatin was the most prescribed medication [16].

In this study, 69% (164) of the individuals had elevated lipid levels. Among them, 24% (58) of participants had one abnormal lipid parameter and 70% (166) had several abnormal lipid parameters. Borle et al. discovered that 28% (14) of participants exhibited isolated lipid profile abnormalities, while 58% (29) had combined and mixed lipid profile abnormalities [8]. According to Hyassat et al., 62.48% (493) of participants had combined and mixed deranged lipid profiles and 27.8% (220) had isolated lipid profile abnormality [12]. A study by Sarfraz et al. also observed that 34% (68) participants had isolated and 66% (132) had combined and mixed dyslipidemia [17]. In the present study, low level of HDL-C was found in 35% (82), hypertriglyceridemia in 44% (104), elevated levels of LDL in 60% (142) and hypercholesterolemia were found in 26% (61), respectively. Comparable results were observed by Dayakar et al. [18].

Gender, age, occupation, hypertension, family history of diabetes, and disease duration exhibited no statistically significant association with the incidence of dyslipidemia in the present study. Also, no statistical significance was found between hypertension and abnormal lipid profiles (p=0.60). Participants with hypertension had a 1.2 times greater likelihood of having high lipid levels (OR=1.2; CI=0.60-2.38; p=0.59). In contrast to a study conducted by Bekele et al. where hypertension had 1.3 times more chance of having dyslipidemia (AOR=1.331; CI=0.436-4.062; p=0.016), and Haile et al. found that participants with hypertension had 2.65 times more chance of having dyslipidemia (AOR-2.65; CI-1.4-4.9; p=0.01) [9,10].

A family history of type 2 diabetes in this study did not statistically significantly correlate with abnormal lipid profiles (p=0.42). The odds of having abnormal lipid profiles were also 1.17 times higher in persons with a family history (OR=1.17; CI=0.62-2.20; p=0.62). Bekele et al. reported similar findings, finding that participants with a family history had a 2.1 times greater likelihood of having high lipid levels (AOR=2.1; CI=0.454-9.877; p=0.339) [9]. HbA1c exhibited no statistically significant connection with abnormal lipid profiles in this study (p=0.79). Participants with poor glycemic control were more likely to have high lipid levels (OR=1.00; CI=0.89-1.14; p=0.98). Kebede et al. found that patients with poor glycemic control were 1.3 times more likely to have abnormal lipid profiles (OR=1.3; CI=0.8-1.9; p>0.05) [11].

BMI was shown to be strongly linked with abnormal lipid profiles in this study (p=0.004). It was also observed that those who were overweight or obese had 1.07 times greater likelihood of having high lipid levels (OR=1.07; CI=0.98-1.16; p=0.92). A similar substantial correlation was observed by Kebede et al., where obese participants had 3.5 times the risk of having disordered lipid profiles (OR=3.5; CI=1.6-7.9; p 0.001). Ahmmed et al. found that participants who were overweight or obese had a 2.08 times greater likelihood of having high lipid levels (OR=2.08; CI=1.73-2.23; p 0.001) [11,19].

In the present study, drug use was found to have a statistically significant relationship with dyslipidemia (p 0.001). Participants who were not using cholesterol-lowering medication had a 5.38 times greater likelihood of having abnormal lipid profiles than those who were (OR=5.38; CI=2.82-10.28; p 0.001). There is insufficient evidence or previous research to support the findings.

Limitations of the study

In this study the sample size was small. Participants in this study belonged to a small geographical area and were from a single centre. High-risk participants from the cardiology department were not included. Confounding factors such as poor glycemic control and concurrent primary hyperlipidemias that act independently of T2DM were not evaluated. 

## Conclusions

In this study, a high prevalence of increased lipid levels was observed among diabetic patients. Also, poor T2DM control was found to contribute significantly to high lipid levels, with other confounders such as primary hyperlipaemias playing a role in the overall process. This study has highlighted the need for a collaborative approach between healthcare providers and patients toward the holistic goal of the management of T2DM and associated comorbidities.
